# Acaricidal activity of *Cinnamomum cassia* (Chinese cinnamon) against the tick *Haemaphysalis longicornis* is linked to its content of (*E*)-cinnamaldehyde

**DOI:** 10.1186/s13071-021-04830-2

**Published:** 2021-06-22

**Authors:** Chuks F. Nwanade, Min Wang, Tianhong Wang, Xiaoyu Zhang, Can Wang, Zhijun Yu, Jingze Liu

**Affiliations:** 1 Hebei Key Laboratory of Animal Physiology, Biochemistry and Molecular Biology, College of Life Sciences, Hebei Normal University, Shijiazhuang, 050024 China; 2 Department of Biochemistry and Biology, Basic Medical College, Hebei University of Chinese Medicine, Shijiazhuang, 050200 China

**Keywords:** *Cinnamomum cassia*, Extract, Essential oil, (*E*)-cinnamaldehyde, *Haemaphysalis longicornis*, Non-target organism

## Abstract

**Background:**

The tick *Haemaphysalis longicornis* (Neumann) is a well-known vector of numerous pathogens of veterinary and medical importance. Various control strategies, including the use of synthetic pesticides, have been developed to control this tick species. However, demand for effective and safe alternative pesticides is increasing due to the adverse effects associated with the intensive and injudicious use of synthetic pesticides, which include undesirable effects on non-target species and environmental pollution. Hence, the acaricidal activity of the extract and the essential oil of *Cinnamomum cassia* (Chinese cinnamon) and their major components, and the underlying mechanisms of this activity, were evaluated against unfed larvae and nymphs of *H*. *longicornis*.

**Methods:**

The components of the extract and essential oil of *C. cassia* were determined by gas chromatography-mass spectrometry, and their larvicidal and nymphicidal activity were evaluated using the larval and nymphal packet test. The underlying detoxification mechanism was elucidated by targeting* in vivo* esterase and monooxygenase activity, and the toxicological effect was assessed on non-target *Tenebrio molitor* and *Harmonia axyridis* by topical application in open Petri dishes.

**Results:**

(*E*)-cinnamaldehyde was the predominant component of the extract (50.79%) and essential oil (89.95%). The 50% lethal concentration (LC_50_) for larvae and nymphs treated with the extract was 11.56 and 49.18 mg/mL, respectively. The essential oil, (*E*)-cinnamaldehyde and fenvalerate exhibited acaricidal activity, with LC_50_ values of 3.81, 3.15, and 0.14 mg/mL, respectively, against the larvae, and 21.31, 16.93, and 1.89 mg/mL, respectively, against the nymphs. (*E*)-cinnamaldehyde significantly increased esterase and monooxygenase activity in both larvae and nymphs. Unlike fenvalerate, *C*. *cassia* essential oil and (*E*)-cinnamaldehyde did not cause mortality of *T*. *molitor* or *H*. *axyridis* adults.

**Conclusions:**

This study demonstrates that *C*. *cassia* essential oil and (*E*)-cinnamaldehyde have the potential to be developed into botanical-based larvicidal and nymphicidal agents for tick control.

**Graphical abstract:**

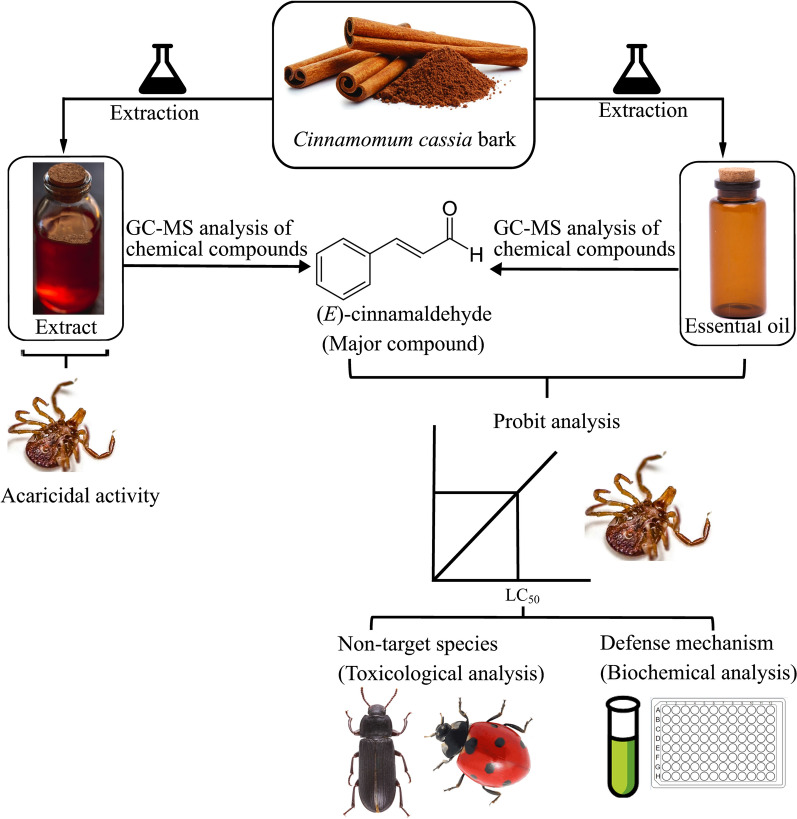

## Background

The long-horned tick *Haemaphysalis longicornis* Neumann (Acari: Ixodidae) is an obligate hematophagous ectoparasite native to East Asian countries, and was recently found to be established in several states of the USA [1]. In recent years, control of this tick has attracted much attention because of its ability to acquire and transmit numerous zoonotic pathogens, including severe fever with thrombocytopenia syndrome virus [2, 3]. In addition, it is a major pest of livestock in Australia and New Zealand, and infestation, which can cause exsanguination, can lead to economic losses related to reduced growth and production [4]. Various control strategies, including the use of synthetic pesticides, have been developed to control tick species including *H*. *longicornis* [5]. However, the demand for effective and safe alternative pesticides is increasing due to the adverse effects, including environmental pollution and undesirable effects on non-target species, associated with the intensive and injudicious use of synthetic pesticides [6]. Plant-based products including essential oils and extracts provide good alternatives because they are effective against a wide variety of pests and have little or no toxicity against non-target species [7, 8]. *Cinnamomum* (Lauraceae) is among the plant genera with promising insecticidal activity [9]. This genus is composed of about 250 species, including the economically important *Cinnamomum cassia* Presl [10].

*C*. *cassia* is an evergreen aromatic plant species native to tropical and subtropical regions including China [11]. It is an important material in Chinese traditional medicine for the treatment of various ailments such as dysmenorrhea and menoxenia [11]. *C*. *cassia* also has numerous pharmacological properties that are of great importance to the food and pharmaceutical industries. For example, *C*. *cassia* essential oil has been demonstrated to be an effective natural antibacterial agent against Shiga toxin-producing *Escherichia coli*, an important foodborne pathogen [12]. Another potential industrial application of *C*. *cassia* is as an antioxidant agent because of its radical scavenging ability [13]. *C*. *cassia* has several other important pharmacological properties, including anticholesterol and antidiabetes activities [14]. Despite its medicinal uses and potential for use in industry, its potential for application in tick management has scarcely been examined.

In the present study, the extract and essential oil of *C. cassia* bark, and their major component, (*E*)-cinnamaldehyde, which were characterized by gas chromatography-mass spectrometry, and the (synthetic) pyrethroid, fenvalerate, were evaluated* in vitro* against unfed larvae and nymphs of *H*. *longicornis*. The larvae and nymphs are important immature stages of *H*. *longicornis* as they have been implicated as vectors of tick-borne pathogens, including *Rickettsia rickettsii*, the agent of Rocky Mountain spotted fever [15]. Therefore, they are considered strategic targets in tick control programs [16, 17]. Furthermore, to elucidate their possible detoxification mechanisms, the effects of the essential oil and (*E*)-cinnamaldehyde [with low 50% lethal concentration (LC_50_) values in the* in vitro* test] on the biochemical parameters of larvae and nymphs of *H*. *longicornis* were tested by targeting* in vivo* esterase and monooxygenase activity. In ticks, esterase and monooxygenase are common detoxification enzymes against xenobiotic compounds [18]. Finally, to evaluate their environmental safety, the toxicological effects of the essential oil and (*E*)-cinnamaldehyde were evaluated on the following non-target terrestrial invertebrates: *Tenebrio molitor* (Coleoptera: Tenebrionidae), a beneficial insect of ecological and nutritional importance [19, 20]; and *Harmonia axyridis* (Coleoptera: Coccinellidae), a biocontrol agent of aphids, scales and other phytophagous pests [21].

## Methods

### Preparation of extracts and source of essential oil

The bark of *C*. *cassia* was obtained commercially in September 2019, from Yulin, Guangxi, China (22.625248°N, 110.18473°E), and identified in the Herbarium of College of Life Sciences, Hebei Normal University, Shijiazhuang, China, from voucher specimen 20,006,001. The dried and pulverized bark of *C*. *cassia* was macerated with 100% methanol, as described by Rawa et al. [22]. The supernatants were collected using a Buchner funnel. The resulting filtrates were dried under reduced pressure at 40 °C using a vacuum rotary evaporator. Extracts were stored at 4 °C in an airtight brown bottle until tested for acaricidal activity. *C*. *cassia* bark essential oil [Generally Regarded as Safe for human consumption (US Food and Drug Administration)] was obtained from a local supplier (Yu Cheng product workshop) in Chaozhou, Guangdong, China. This supplier extracts essential oils on a small scale by hydrodistillation.

### Determination of the components of *C*. *cassia* extract and essential oil

Components of *C*. *cassia* extract and essential oil were determined on a gas chromatography-mass spectrometer (model 7890A-5975C; Agilent Technologies, USA). Chromatographic separation was achieved using a DB-5MS capillary column (30 m × 0.25 mm × 0.25 µm). The carrier gas was helium at 1 mL/min flow rate, and the spilt ratio 10:1. The temperature conditions were 60 °C for 2 min, increased to 150 °C for 1 min, and finally to 280 °C for 5 min. The mass spectrometer was bombarded with an electron impact ionization source, and the electron energy was 70 eV. The inlet temperature was 280 °C and the ion source temperature was 230 °C. The mass scanning range was 40–550 m/z. Compounds were identified using the Standard Reference Database of the National Institute of Standards and Technology.

### Chemicals

Analytical grade methanol was purchased from Baishi Chemicals (Tianjin, China). (*E*)-cinnamaldehyde (98%; lot no. I1918074), a clear yellow liquid, was purchased from Aladdin Biochemical (Shanghai, China). Fenvalerate (Hubei Danongren Biological Technology), used as a sample synthetic (commercial) acaricide, was purchased from a local market. Esterase, monooxygenase, and protein assay kits were purchased from Shanghai Enzyme-linked Biotechnology (Shanghai, China).

### Test organisms

Unfed adult ticks of *H. longicornis* were collected from vegetation by tick dragging at Xiaowutai National Nature Reserve Area (40°03′03″N, 115°23′15″E), Zhangjiakou, Hebei Province, China. Unfed adult ticks were placed on the ears of New Zealand white rabbits and the resulting engorged females were maintained in an incubator [26 ± 1 °C, 85 ± 5% relative humidity (RH), 16:8 h (light:dark; L:D) photoperiod] for oviposition. The larvae that hatched during a period of 14 days and the nymphs that molted during a period of 14 days were used for subsequent tests.

*T*. *molitor* larvae were collected from a commercial breeder in Zhengzhou, Henan, China. They were first reared to the pupal and then to the adult stage on a diet of wheat bran and potato slices in plastic containers (18 × 11 × 11 cm), as described by George et al. [19], under laboratory conditions [25 ± 1 °C, 70 ± 5% RH, 16:8-h photoperiod (L:D)]. Adults 6–10 days after eclosion, and larvae (> 15 mm), were used in this study [23]. *H*. *axyridis* larvae were commercially obtained (Jiangsu, China), and reared according to Benelli et al. [8]. Larvae were fed aphids *ad libitum* and maintained in an incubator [25 ± 1 °C, 70 ± 5% RH, 16:8-h photoperiod (L:D)], and the resulting adults (3–7 days old) were used in this study.

### Acaricidal activity

Acaricidal activity against the larvae and nymphs of *H*. *longicornis* was determined using larval and nymphal packet tests. First, the bark extract of *C*. *cassia*, essential oil of *C*. *cassia*, and (*E*)-cinnamaldehyde were diluted with 70% methanol. There is little or no toxicity of 70% methanol when it is used as a solvent in the packet test* in vitro* (filter papers soaked with the test compounds were allowed to dry before use in a closed packet containing ticks) [see details of the larval packet test (LPT) below]. In addition, previous studies have shown that analytical grade methanol (undiluted) has little or no toxicity against tick species, and it has been suggested as a suitable solvent for dissolving plant extracts when testing acaricidal activity [24]. Then a range finding test was carried out to determine the appropriate test concentrations. For larvae, five concentrations were used, ranging from 5.00 to 20.00 mg/mL for the extract, 2.75–5.00 mg/mL for the essential oil, 2.75–3.70 mg/mL for (*E*)-cinnamaldehyde, and 0.025–0.750 mg/mL for fenvalerate. For nymphs, five to six concentrations were used, ranging from 40.00 to 60.00 mg/mL for the extract, 10.00–30.00 mg/mL for the essential oil, 5.00–30.00 mg/mL for (*E*)-cinnamaldehyde, and 0.38–4.50 mg/mL for fenvalerate.

The LPT was carried out according to the* in vitro* test recommended by the Food and Agriculture Organization of the United Nations [25], as described by Godara et al. [26]. Briefly, filter papers (7.5 × 9.0 cm, Whatman No. 1; Whatman, Maidstone, UK) were soaked with 1 mL of the dilutions. The treated filter papers were allowed to dry and were then folded into packets and sealed on the side with clips. One hundred larvae were introduced into each packet before sealing the top of with clips. The packets containing the larvae were kept in an incubator (26 ± 1 °C and 85 ± 5% RH). After 24 h, the packets were examined for larval mortality. Distilled water and 70% methanol served as the negative control group, and fenvalerate diluted with distilled water, as specified on the product label, served as the positive control. Each treatment and control group was replicated four times. A similar LPT was used for unfed nymphs, with ten nymphs placed into each packet; each treatment and the negative control group (distilled water and 70% methanol) and positive control group (fenvalerate) was replicated five times.

### Effect (LC_50_) on non-target *T*. *molitor*

**Fig. 1:**
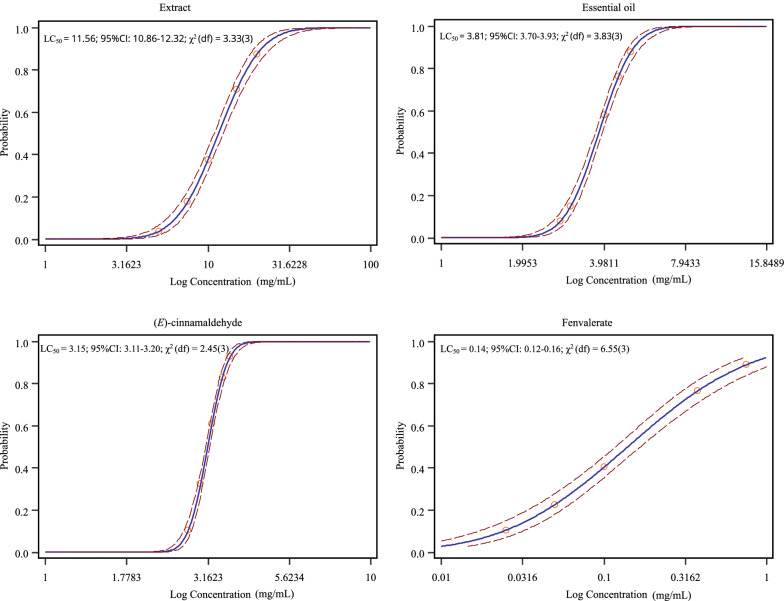
Concentration–response curves of *Haemaphysalis* *longicornis* larvae.* Solid lines* represent the estimated mean.* Dotted lines* represent 95% confidence intervals (*CI*; lower and upper) around the mean. *LC*_*50*_ Lethal concentration required to kill 50% of the population at 24 h

**Fig. 2:**
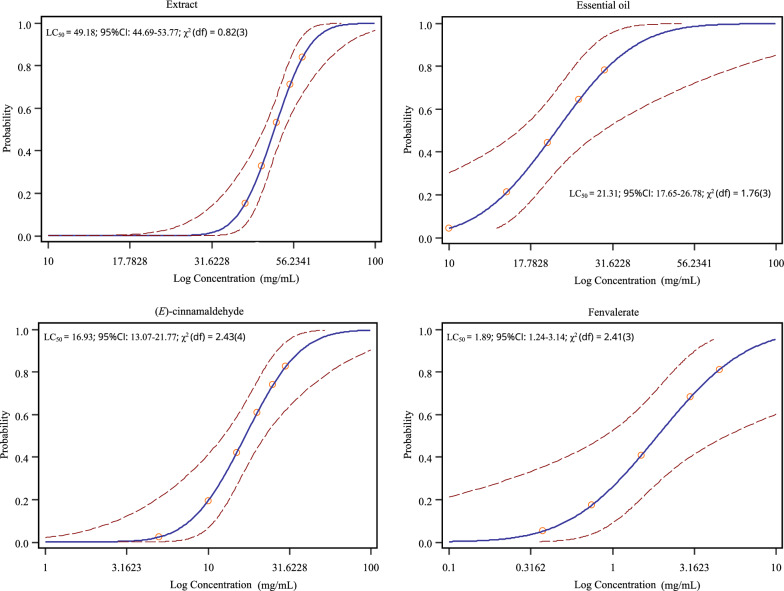
Concentration–response curves of *H*. *longicornis* nymphs.* Solid lines* represent the estimated mean.* Dotted lines* represent 95% CI around the mean. For abbreviations, see Fig. 1

The essential oil and (*E*)-cinnamaldehyde of *C*. *cassia* were selected due to their low LC_50_ values against unfed larvae and nymphs of *H*. *longicornis* (Figs. 1, 2). Adults of *T. molitor* were exposed to LC_50_ of *C*. *cassia* essential oil (3.81 and 21.31 mg/mL, respectively, for larvae and nymphs of *H*. *longicornis*) and (*E*)-cinnamaldehyde (3.15 and 16.93 mg/mL, respectively) (Figs. 1, 2) using the method described by Pedersen et al. [23]. Exposure was performed by topical application in open Petri dishes (diameter, 9 cm). Briefly, the abdomen was held with a soft tweezer, causing the beetle to lower its head, which exposed the soft part between the pronotum and elytra. Each adult received a 1-µL droplet of the test solutions using a micropipette (Eppendorf, Hamburg, Germany). Likewise, larvae received a 1-µL droplet of the test solutions in the area that was proportionally equal in proximity to the head. Adults and larvae of *T*. *molitor* exposed to LC_50_ of fenvalerate (0.14 and 1.89 mg/mL, respectively, for larvae and nymphs of *H*. *longicornis*), as estimated in the toxicity bioassay, were used as a positive control. Negative control groups included adults and larvae exposed to 70% methanol and distilled water. Treated adults and larvae were transferred to clean Petri dishes (ten beetles per dish), and fed with wheat bran and potato slices. All dishes were maintained in the incubator [25 ± 1 °C, 70 ± 5% RH, 16:8-h photoperiod (L:D)]. The numbers of dead larvae and adults of *T*. *molitor* were determined 48 h after treatment. *T*. *molitor* that did not respond to the probe were considered dead. Each treatment and control group was replicated five times.

### Effect (LC_50_) on non-target *H*. *axyridis*

The effects of the LC_50_ for larvae and nymphs of *H*. *longicornis* of *C*. *cassia* essential oil (3.81 and 21.31 mg/mL, respectively) and (*E*)-cinnamaldehyde (3.15 and 16.93 mg/mL, respectively) on adult *H*. *axyridis* were assessed by using a minor modification of the method described by Youn et al. [27]. In the present study, 1 µL of the test solutions was topically applied to adults at the junction between the pronotum and elytron using a micropipette (Eppendorf). Adults of *H*. *axyridis* exposed to LC_50_ of fenvalerate for larvae and nymphs of *H*. *longicornis* (0.14 and 1.89 mg/mL) were used as positive controls. Negative control groups included adults exposed to 70% methanol and distilled water. Treated adults were transferred to clean Petri dishes (ten adults per dish), fed with *Metopolophium dirhodum,* and maintained in an incubator [25 ± 1 °C, 70 ± 5% RH, 16:8-h photoperiod (L:D)]. The number of dead *H*. *axyridis* was determined 48 h after treatment [7], with adults that did not respond to a probe considered dead. Each treatment and the control groups were replicated five times.

### Biochemical analysis

The* in vivo* enzyme activities were measured for unfed larvae and nymphs of *H*. *longicornis* exposed to LC_50_ of the essential oil (3.81 and 21.31 mg/mL, respectively) and (*E*)-cinnamaldehyde (3.15 and 16.93 mg/mL, respectively) (Figs. 1, 2). Larvae and nymphs exposed to LC_50_ of fenvalerate (0.14 and 1.89 mg/mL, respectively) (Figs. 1, 2) were used as a positive control. Negative control groups included larvae and nymphs exposed to 70% methanol and distilled water. The essential oil and (*E*)-cinnamaldehyde were selected due to their low LC_50_ values against unfed larvae and nymphs of *H*. *longicornis* (Figs. 1, 2). About 20 mg of the larvae and nymphs that survived after treatments in the packet assays was homogenized in 400 µL pre-cooled phosphate-buffered solution (0.1 M, pH 7.2). The homogenate was centrifuged at 3000 × *g* at 4 °C for 10 min. The resulting supernatant was removed and used for esterase and monooxygenase assays using the corresponding ELISA kit (Shanghai Enzyme-linked Biotechnology), and for the protein assay using bicinchoninic acid protein assay kit (Shanghai Enzyme-linked Biotechnology), following the manufacturer's instructions. For each tested concentration and control group, there were three replicates.

### Statistical analyses

Statistical analyses were performed using SPSS software (v20.0; IBM, Armonk, NY). The normality of residuals was first verified using the Shapiro–Wilk test. Then, ANOVA and Tukey’s test were used to determine the significance of the data of the biochemical analyses. Kruskal–Wallis and Student-Newman-Keuls post hoc test were used to determine the significance of the percentage mortality of the non-target species. Mortality of *H*. *longicornis* larvae in the negative control (70% methanol only) was below 5%, thus no correction of mortality was necessary [25]. LC_50_ were determined by performing a probit analysis on the tick mortality data [28] using SPSS (v20.0) and MedCalc (v19.6.4; MedCalc, Ostend, Belgium) software.

## Results and discussion

### Chemical composition

In the *C*. *cassia* extract, a total of 14 compounds representing 97.39% of the total extract were identified. The major constituent of this extract was (*E*)-cinnamaldehyde (50.79%). The percentage compositions of the remaining 13 compounds ranged from 0.97 to 15.83% (Table 1). In *C*. *cassia* essential oil, 16 compounds were identified, accounting for 97.11% of the total composition. Specifically, the major constituent was (*E*)-cinnamaldehyde (89.95%). The percentage abundance of the remaining 15 compounds ranged from 0.13 to 1.68% (Table 1).

**Table 1:** The compounds obtained from the bark extract and essential oil of *Cinnamomum cassia*

S/N	Retention time	Area %	Compounds		Area %	Retention time
			Extract	Essential oil		
1	6.748	11.99	3-Methoxy-3-phenylpropanal	Tetrahydro-2-furanol	0.42	3.230
2	6.951	*50.79*	(*E*)-*cinnamaldehyde*	Butyrolactone	0.16	4.999
3	7.582	2.83	Copaene	2-Furanmethanol	1.39	5.124
4	8.016	1.78	Cinnamyl acetate	Benzaldehyde	0.26	5.688
5	8.183	15.83	(*E*)-2-hydroxycinnamic acid	(*Z*)-cinnamaldehyde	0.52	8.240
6	8.431	2.53	*α*-Muurolene	(*E*)-*cinnamaldehyde*	*89.95*	8.809
7	8.559	3.16	*β*-Cadinene	Copaene	0.18	9.433
8	8.601	1.21	*Cis*-calamenene	*Trans*-cinnamic acid	1.09	9.777
9	8.642	1.83	2-Methoxycinnamaldehyde	Cinnamyl ester	0.40	9.842
10	8.671	0.97	Cubenene	Coumarin	0.33	9.919
11	9.399	1.14	Syringaldehyde	*γ*-Muurolene	0.15	10.092
12	9.399	1.14	*T*-muurolol	Cadina-1(10),4-diene	0.13	10.353
13	11.013	1.03	Palmitic acid	2-Methoxycinnamaldehyde	1.68	10.430
14	11.876	1.16	Linoleic acid	Benzene	0.18	10.55
15				2-Methyl-1-naphthalenol	0.14	10.727
16				1-Phenyl-hexa-1,5-dione	0.13	11.071

*S*/*N* Signal-to-noise ratio

The presence of (*E*)-cinnamaldehyde as the predominant component in the extract and essential oil from *C*. *cassia* bark in the present study is in accordance with results reported in other studies [29, 30]. However, the chemical composition of the extract and essential oil from *C*. *cassia* bark is affected by many factors including methods of extraction, and the different agroecological conditions during the growth and development of the plant [30]. As a result, several species of *C*. *cassia* have been reported with different percent contents of (*E*)-cinnamaldehyde [29, 30]. For example, in the study of Deng et al. [29], (*E*)-cinnamaldehyde was the predominant component and accounted for about 72.23% of *C*. *cassia* bark essential oil. In a recent study, Liang et al. [30] found that (*E*)-cinnamaldehyde (62.96%) was a major component of *C*. *cassia* bark extract. In addition, in other species of *Cinnamomum*, (*E*)-cinnamaldehyde is also a major component, and accounted for about 66.43% of *Cinnamomum* *zeylanicum* bark essential oil [31]. In the present study, the percent content of (*E*)-cinnamaldehyde in the essential oil (89.95%) was higher than in the extract (50.79%). A possible explanation for variation in the percent content of (*E*)-cinnamaldehyde may be the different methods used for the extraction of the extract and essential oil. Variation in the percent content of (*E*)-cinnamaldehyde can also be due to the geographical origin of the *C*. *cassia* and the method used for the extraction of the extract and essential oil [30].

### Acaricidal activity, effect (LC_50_) on non-target species, and biochemical activity

**Fig. 3:**
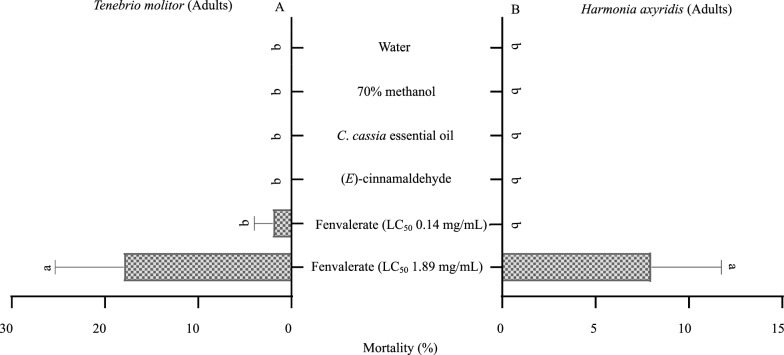
Mortality (%) of *Tenebrio* *molitor* (**A**) and *Harmonia* *axyridis* (**B**). The fenvalerate LC_50_ values were estimated for the larvae and nymphs of *H*. *longicornis* by the packet test. Data are presented as mean ± SE (*n* = 5).* Different letters* indicate significant difference (Kruskal–Wallis/Student-Newman-Keuls, *p* < 0.01). For abbreviations, see Fig. 1

The acaricidal activity was evaluated by the packet test for unfed larvae and nymphs of *H*. *longicornis*. After treatment of unfed larvae and nymphs of *H*. *longicornis* for 24 h, *C*. *cassia* essential oil, with LC_50_ values of 3.81 and 21.31 mg/mL, respectively, and (*E*)-cinnamaldehyde, with LC_50_ values of 3.15 and 16.93 mg/mL, respectively, exhibited greater acaricidal activity compared with *C*. *cassia* extract, with LC_50_ values of 11.56 and 49.18 mg/mL, respectively (Figs. 1, 2). However, the efficacy of the essential oil and (*E*)-cinnamaldehyde was lower compared with that of fenvalerate (synthetic acaricide), with LC_50_ values of 0.14 and 1.89 mg/mL against the larvae and nymphs, respectively (Figs. 1, 2). Despite these results, *C*. *cassia* essential oil and (*E*)-cinnamaldehyde can be considered as potential candidates for the development of larvicidal and nymphicidal agents for tick control. This conclusion is justified from a toxicological and a mechanistic perspective. For instance, from a toxicological point of view, it is important not only to assess the efficacy of new plant-based pesticides on target species but also to estimate any effect on non-target species [7]. Herein, we evaluated the effect of *C*. *cassia* essential oil and (*E*)-cinnamaldehyde on the mortality of non-target species, i.e. *T*. *molitor* and *H*. *axyridis*. *T*. *molitor* is presently being promoted as a beneficial insect, as it is capable of degrading polystyrene and plastic waste, and may play an important role in litter decomposition [19, 20]. In addition, *T*. *molitor* larvae are often used as pet food, and offer a promising alternative protein source for human and animal nutrition [20]. Besides this, predatory behavior of *T*. *molitor* on ticks has been reported [32]. *H*. *axyridis* is a biocontrol agent of pests including aphids, scales, and other phytophagous pests, and plays an important role in regulating the populations of these pests [21]. Topical application of the essential oil (tested concentration: LC_50_ 3.81 and 21.31 mg/mL for unfed larvae and nymphs of *H*. *longicornis*) or (*E*)-cinnamaldehyde (tested concentration: LC_50_ 3.15 and 16.93 mg/mL, respectively) did not cause mortality in *T*. *molitor* or *Harmonia* *axyridis* adults (Fig. 3A, B), which may indicate that these compounds are environmentally safe. Generally, essential oils and their active compound(s) are not toxic to non-target organisms [7, 8], in accordance with our results. For example, essential oil from *Stevia rebaudiana* did not cause any significant mortality in *H*. *axyridis* larvae or adults, and had no adverse effect on *Eisenia fetida* earthworm adults [8].

However, topical application of fenvalerate (tested concentration: LC_50_ 1.89 mg/mL for the nymphs of *H*. *longicornis*) resulted in significant mortality in adult *T*. *molitor* [18.00 ± 7.35;* F*_(4,20)_ = 15.444, *p* = 0.000] and *H*. *axyridis* [8.00 ± 3.74;* F*_(4,20)_ = 5.952, *p* = 0.003] when compared with the control (Fig. 3A, B), which indicates that fenvalerate may inhibit their performance as beneficial organisms and biocontrol agents. Furthermore, there was no mortality in the larvae of *T*. *molitor* treated with fenvalerate (tested concentrations: LC_50_ 0.14 and LC_50_ 1.89 mg/mL, for larvae and nymphs of *H*. *longicornis*) during the 48-h observation period, thus these data were omitted from all calculations and comparisons. Pedersen et al. [23] found that *T*. *molitor* adults were more sensitive to exposure to the pyrethroid α-cypermethrin than their larvae. It is possible that the size of an insect and its structure may affect the amount of a chemical that enters its body [8, 23]. Generally, synthetic insecticides including pyrethroids have a negative effect on non-target species [7, 8]. For example, treatment with α-cypermethrin at 1.6 × 10^−6^ mL/L caused about 50% mortality in non-target *H*. *axyridis* adults [8].

It is important to note that the results of the non-target assay should be interpreted with caution, given the different experimental designs, including the non-uniform methods (LPT verse topical application) used to assess toxicity on the target and non-target species, which may have caused *Haemaphysalis* *longicornis*, *T*. *molitor* and *Harmonia* *axyridis* to react differently to the test substances. Moreover, the toxicity of a substance usually depends on the amount acquired by the test organism [8, 23]. Thus, the lack of toxicity of *C*. *cassia* essential oil and (*E*)-cinnamaldehyde in non-target *T*. *molitor* and *H*. *axyridis* could have been due to their exposure to lower concentrations than the ticks, as a result of the different* in vitro* assays used. However, our observations in this study are significant in that they provide an indication of the potential effects of *C*. *cassia* essential oil and (*E*)-cinnamaldehyde on non-target *T*. *molitor* and *H*. *axyridis*, and provide the basis for the design of future studies which will take into account, among other factors, the disparities between the experimental designs used in this present study.

**Fig. 4:**
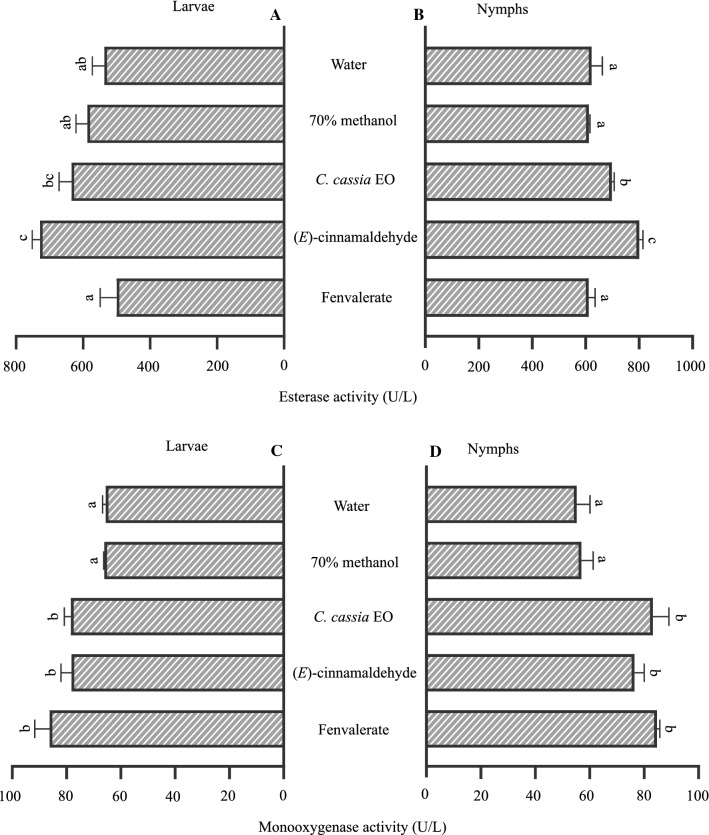
**A–D** Esterase and monooxygenase activities of larvae and nymphs of *H*. *longicornis.* Data are presented as the mean ± SE (*n* = 3).* Different letters* indicate a significant difference (Tukey’s test, *p* < 0.001).* EO* Essential oil

Elucidating the underlying detoxification mechanism can provide important clues for the development of botanical-based acaricides. In the present study, both the essential oil of *C*. *cassia* and (*E*)-cinnamaldehyde significantly affected the activities of detoxification enzymes in ticks, including those of esterase and monooxygenase. For example, in contrast to the fenvalerate treatment, esterase activity was significantly increased in larvae [Fig. 4A;* F*_(4,10)_ = 17.549, *p* = 0.000] and nymphs of *H*. *longicornis* [Fig. 4B;* F*_(4,10)_ = 39.363, *p* = 0.000] exposed to (*E*)-cinnamaldehyde (tested concentration: LC_50_ 3.15 and 16.93 mg/mL, respectively) when compared with the control. Likewise, esterase activity significantly increased when the nymphs were exposed to *C*. *cassia* essential oil (tested concentration: LC_50_ 21.31 mg/mL) (Fig. 4B). On the other hand, similar to the effects of fenvalerate, monooxygenase activity was significantly increased in the larvae [Fig. 4C;* F*_(4,10)_ = 22.346, *p* = 0.000] and nymphs [Fig. 4D;* F*_(4,10)_ = 33.181, *p* = 0.000] after treatment with LC_50_ of *C*. *cassia* essential oil and (*E*)-cinnamaldehyde. Esterase and monooxygenase are involved in the metabolic detoxification of pesticides [33]. In ticks, they play an important role in the development of resistance to synthetic acaricides [18, 34]. For example, resistance to synthetic pyrethroids was found to be significantly correlated with increase esterase and monooxygenase activities in *Rhipicephalus microplus* [34]. It has also been demonstrated that the inhibition of detoxification enzymes, including esterase, led to an increase in the mortality of a resistant strain of *R. microplus* [35]. The increased expression of esterase and monooxygenase in the present study may indicate their involvement in the detoxification response against *C*. *cassia* essential oil and (*E*)-cinnamaldehyde. However, additional studies are needed to test different times of exposure, since esterase and monooxygenase activities may decrease with time [33], and to explore the contribution of other metabolic enzymes such as acetylcholinesterase and glutathione S-transferases to defense mechanisms. It is important to note that the total protein content of *H*. *longicornis* after treatment with *C*. *cassia* essential oil, (*E*)-cinnamaldehyde and fenvalerate ranged from 0.98 to 1.05 mg/mL for the larvae and 0.96–1.13 mg/mL for the nymphs, and treatment with the essential oil, (*E*)-cinnamaldehyde and fenvalerate did not induce significant changes in the total protein of either.

In addition, based on the present results, the efficacy of *C. cassia* essential oil in terms of LC_50_ values, i.e. lower concentrations compared with those of the extract (Figs. 1, 2), may be attributed to the high percent content of the main component, (*E*)-cinnamaldehyde. (*E*)-cinnamaldehyde is the key component of *C. cassia* bark responsible for its insecticidal and acaricidal activity [36]. When tested as a pure compound, (*E*)-cinnamaldehyde was effective against the larvae and nymphs of *Rhipicephalus sanguineus*, with 100% mortality at 2.55 and 10 μL/mL, respectively [37]. A similar observation was recorded for the larvae of *Amblyomma cajennense* at a (*E*)-cinnamaldehyde concentration of 5.0 μL/mL [37]. Likewise, (*E*)-cinnamaldehyde was effective with 100% mortality at a concentration of 5 μL/mL against *R*. *microplus* and *Dermacentor nitens* larvae [38]. Acaricidal activity of (*E*)-cinnamaldehyde was also observed against the larvae of *Amblyomma sculptum* (LC_50_ 1.40 mg/mL) and *D*. *nitens* (LC_50_ 1.68 mg/mL) [39]. Besides its efficacy against ticks, (*E*)-cinnamaldehyde was found to be an efficient mosquito larvicidal against *Aedes albopictus*, *Culex quinquefasciatus* and *Armigeres subalbatus*, with LC_50_ values below 50 µg/mL [40].

Compared with other essential oils, for example, essential oil of *Cymbopogon citratus* (LC_50_ values of 28.06 and 28.18 mg/mL for larvae and nymphs of *H*. *longicornis*, respectively) [41], *C*. *cassia* bark essential oil tested in this study showed greater acaricidal activity against the larvae and nymphs of *H*. *longicornis*. The insecticidal activity of *C*. *cassia* essential oil has also been reported for other target species, e.g. against the nymphs of *Ricania* sp., with an LC_50_ value of 37.66 mg/mL after exposure for 72 h [42]. In addition, the insecticidal activity of *C*. *cassia* extract has been reported for pests of stored products including *Tribolium castaneum* and *Lasioderma serricorne*, with LD_50_ values of 3.96 and 23.89 μg/adult, respectively [43]. To our knowledge, the present study is the first report on the acaricidal activity of *C*. *cassia* extract and essential oil against vector ticks. The essential oils obtained from other species of *Cinnamomum* have also been studied against tick species. For example, the larvicidal activity of *Cinnamomum* *zeylanicum* essential oil was evaluated against *R*. *microplus* larvae, with an LC_50_ of 0.086% [44]. Apart from acaricidal activity, *Cinnamomum* *verum* essential oil was found to exhibit larvicidal activity against fourth-instar *Cx. quinquefasciatus* (LC_50_ 40.7 μL/L) and adulticidal (LC_50_ 42 μg/adult) activity against *Musca domestica* [9].

Cinnamon oil is considered safe by the US Food and Drug Administration and is exempted from toxicity data requirements by the US Environmental Protection Agency [36, 42]. However, there are potential safety concerns regarding cinnamaldehyde. For example, cinnamaldehyde at certain exposure concentrations reduces adenosine triphosphate levels and disrupts mitochondrial function in human bronchial epithelial cells [45]. Thus, during* in vivo* and/or field trials of *C*. *cassia* products, concentration–response curves should be determined. These can be used to identify lethal and non-lethal concentrations for ticks and mammalian hosts. Additionally, application of *C*. *cassia* products directly to the host may provide better protection from tick infestation, since hosts can be kept in enclosures to protect them against environmental factors including weather conditions that could interfere with the efficacy of the applied products [46]. Natural product-based acaricides are sensitive to environmental factors, thus, they do not persist for long in the environment [47].

## Conclusions

The *C*. *cassia* products tested in the present study, especially the essential oil and (*E*)-cinnamaldehyde, exhibited good acaricidal activity against larvae and nymphs of *Haemaphysalis* *longicornis*, whereas no toxic effect was found against the terrestrial invertebrates *T*. *molitor* and *Harmonia* *axyridis*, which suggests that they may not be harmful to non-target terrestrial invertebrates when used as acaricides. The defense response of the larvae and nymphs of *H*. *longicornis* to *C*. *cassia* essential oil and (*E*)-cinnamaldehyde may be partly related to the increased synthesis of esterase and monooxygenase. In addition, the acaricidal activity of *C*. *cassia* essential oil or extract is likely related to the biochemical activity of (*E*)-cinnamaldehyde, i.e. the increased expression of esterase and monooxygenase activity. However, the molecular mechanism underlying the acaricidal activity of *C*. *cassia* warrants further exploration, and future studies on its effects on other non-target species such as soil-dwelling (earthworms) and aquatic organisms are imperative.

## Data Availability

The data used and analyzed during the current study are available from the corresponding author on reasonable request.

## Declarations

### Animal welfare statement

The experimental protocols in this study fully complied with the animal protection law of the People’s Republic of China, and were approved by the Animal Ethics Committee of Hebei Normal University (protocol no. 157031).
